# The potential and risks of FLASH radiotherapy in pediatric patients

**DOI:** 10.1093/neuped/wuaf014

**Published:** 2025-11-19

**Authors:** Jan Schuemann, Bethany Rothwell, Anthony Mascia, Yi Fan, Billy W Loo, Torunn Yock, Kevin X Liu, John Perentesis, Daphne A Haas-Kogan

**Affiliations:** Department of Radiation Oncology, Massachusetts General Brigham, Boston, MA, United States; Division of Oncology, Cancer and Blood Disease Institute, Cincinnati Children’s Hospital, Cincinnati, OH, United States; Department of Radiation Oncology, University of Pennsylvania, Philadelphia, PA, United States; Department of Radiation Oncology & Stanford Cancer Institute, Stanford University School of Medicine, Stanford, CA, United States; Department of Radiation Oncology, Massachusetts General Brigham, Boston, MA, United States; Department of Radiation Oncology, Massachusetts General Brigham, Boston, MA, United States; Department of Radiation Oncology, Dana Farber Cancer Institute, Boston Children’s Hospital, Boston, MA, United States; Division of Oncology, Cancer and Blood Disease Institute, Cincinnati Children’s Hospital, Cincinnati, OH, United States; Department of Radiation Oncology, Massachusetts General Brigham, Boston, MA, United States; Department of Radiation Oncology, Dana Farber Cancer Institute, Boston Children’s Hospital, Boston, MA, United States

**Keywords:** ultra-high dose rate, FLASH, pediatrics, clinical translation

## Abstract

Irradiation at ultra-high dose rates, also termed as ‘FLASH’ radiotherapy, has generated great interest in radiation oncology due to its potential to preferentially spare healthy tissue without compromising tumor control. Over the past decade, studies on the potential benefits of FLASH radiotherapy (FLASH-RT) have grown rapidly, yet the underlying mechanisms of this differential effect remain poorly understood. Nonetheless, compelling preclinical data, particularly the preservation of cognitive function after whole-brain irradiation, suggest that FLASH-RT may significantly improve quality of life, especially for pediatric patients. Early preclinical (large animal) and adult clinical trials are underway, prompting consideration of how and when to translate this modality to pediatric populations. This review summarizes the current state of FLASH-RT, with a special focus on pediatric CNS applications. We discuss disease-specific opportunities and limitations, how biological and clinical differences between adults and children may influence the FLASH effect, and key factors that must be evaluated before safe and effective pediatric translation.

Key points

- Ultra-high dose rate radiation therapy, FLASH-RT, may spare normal tissue while preserving tumor control, which is critical for long-term survivors like pediatric patients.
- We highlight the need for special care, considerations, and limitations for potential translation to pediatric patients, with a focus on CNS tumors.

## Introduction

Pediatric patients present unique challenges in radiotherapy due to their generally favorable clinical prognosis, sensitivity of developing normal tissues to radiation, and long life expectancies. While advances in conformal techniques and proton therapy have reduced the amount of irradiated tissue and improved outcomes, the risk of late effects, including neurocognitive decline, growth impairment, and secondary malignancies, remains a major clinical concern in pediatric oncology.^1^ FLASH radiotherapy (FLASH-RT), a technique delivering ultra-high dose rates (typically ≥40 Gy/s) in a few milliseconds, has emerged over the past decade as a potential game-changer. Preclinical studies suggest that FLASH-RT may spare normal tissue while preserving tumor control, thus increasing the therapeutic ratio, a phenomenon coined the “FLASH effect.”^2^ With growing interest in translating FLASH-RT to clinical use, this technique has been widely reviewed.^3-6^ However, its application in pediatric patients remains largely unexplored.

**Figure 1:**
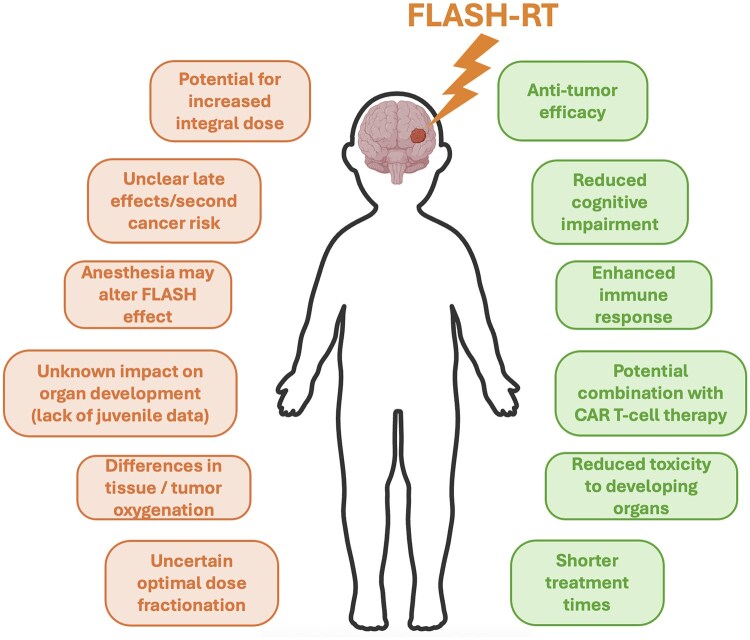
Opportunities (right side) and challenges (left side) of FLASH radiotherapy for treating pediatric patients—for example, in CNS tumors.

In this review, we examine the potential and risks of FLASH-RT for pediatric cancers, with a particular focus on central nervous system (CNS) tumors. We explore what is known from juvenile animal models, highlight unique biological and technical challenges in children, and consider how FLASH-RT might intersect with emerging therapies such as CAR-T cell therapy. Figure 1 highlights the opportunities and challenges of FLASH radiotherapy for treating pediatric patients. A careful assessment of the benefits and risks is essential before this technique can be safely integrated into routine pediatric cancer care.

### FLASH radiotherapy: current status

Since its demonstration in 2014,^2^ FLASH-RT has rapidly emerged as one of the most intensely investigated innovations in radiation oncology.^5^ FLASH-RT has been shown to reduce normal tissue toxicity by ∼20%-50% in multiple preclinical models while maintaining tumor control.^4^^,^^6^ This includes reduced lung injury in mice,^2^^,^^7^ preservation of cognitive function in rodents,^8-13^ and decreased skin toxicity in both mini-pigs^14^ and mice,^15-18^ with comparable tumor control observed across more than 20 tumor types.^4^ Although early studies primarily used electron beams,^19^ the effect has since been shown using protons,^20^ photons,^21^ and heavier ions such as carbon^22^ and helium.^23^

The FLASH effect appears to be influenced by several physical and biological parameters. Dose rate is a key factor: although 40 Gy/s is widely considered as the ‘threshold,’^2^ some studies suggest a more gradient-like threshold with more prominent sparing effects beyond ∼100 Gy/s.^8^ While most studies focus on the average dose rate, some studies suggest that the instantaneous dose rate also matters, with instantaneous dose rates varying widely between radiation modalities.^24^ Other modulating variables may include total dose, fractionation scheme, and beam modality.^24^ Oxygen status has also been shown to play a role, with several studies reporting a diminished or absent FLASH effect in animals with elevated oxygen levels.^25-27^

Despite extensive investigation, the mechanisms underlying the FLASH effect remain unresolved. One early hypothesis, radiolytic oxygen depletion, suggested that ultra-fast dose delivery transiently reduces local oxygen concentrations, thereby rendering normal tissues temporarily more radioresistant.^28-30^ However, this explanation has been questioned, as the magnitude of oxygen depletion observed experimentally may be insufficient to account for the full effect.^4^^,^^31^ Ongoing studies are examining whether FLASH-RT preferentially spares specific tissue regions, such as areas further from capillaries or naturally hypoxic niches.^32^ Other proposed mechanisms center around physicochemical processes, such as radical–radical recombination,^33^ reactive-oxygen-species-mediated cell damage,^25^^,^^34^^,^^35^ and Fenton-chemistry,^36^ as well as differential immune and inflammatory responses from FLASH versus conventional irradiation.^3^^,^^4^^,^^6^ However, no consensus has yet been reached.

Despite this uncertainty, translation to the clinic is already underway. Treatment planning studies, mostly using protons, have explored the feasibility of clinical FLASH-RT implementation,^37^ highlighting significant challenges in consistently achieving the required dose rates (among other parameters) necessary for normal tissue sparing.^38^ To meet these constraints, several novel techniques have been proposed, including the use of transmission proton beams,^39^ patient-specific ridge filters,^40^ and other delivery strategies.^41^^,^^42^ However, planning remains challenging without well-defined parameters that consistently induce the FLASH effect.

Nonetheless, early clinical applications have already begun. Numerous veterinary studies in cats and dogs have demonstrated promising efficacy and tolerability of FLASH-RT.^14^^,^^43-45^ In humans, the first patient was treated with high-dose-rate electrons for a superficial skin tumor just a few years into the field’s emergence.^46^ More recently, several clinical trials have launched. The FAST-01 trial (NCT04592887), a Phase I study using proton transmission FLASH-RT to treat bone metastases in adults, has completed at the Cincinnati Children’s/UC Health Proton Therapy Center, with encouraging safety and feasibility outcomes.^47^^,^^48^ This work is being extended in FAST-02 (NCT05524064), which targets bone metastases in the thorax.^49^ The first conformal proton FLASH-RT trial is being planned at the University of Pennsylvania.^50^ As these early trials are primarily focused on assessing the feasibility of delivering ultra-high dose rate irradiation, they have not included a comparative conventional-dose-rate treatment arm.

Electron FLASH-RT is also being investigated in 2 ongoing trials at Lausanne University Hospital: a Phase I dose-escalation study for malignant melanoma of the skin (NCT04986696) and a Phase II study for basal and squamous cell carcinomas (NCT05724875), both of which are currently enrolling patients. However, so far, no trials have enrolled pediatric patients. The suitability of FLASH-RT for children remains an open, critical question with potential to tackle key challenges in pediatric radiation oncology.

### Pediatric CNS cancers: clinical overview

Radiotherapy is a pillar of curative treatment of pediatric CNS tumors but often carries a high cost of long-term side effects and functional impairment. The unique normal tissue-sparing potential of the FLASH effect may offer a substantial benefit in this context, by reducing toxicity without compromising tumor control.

Pediatric brain tumors are the most common solid tumor in childhood and include many different histologic types, ranging from low-grade glioma and benign meningioma to aggressive tumors, such as high-grade glioma or medulloblastoma.^51^ In addition to systemic therapy and surgery, radiotherapy is frequently an important treatment modality in curing pediatric patients with CNS tumors and for palliation. For many histologies, such as medulloblastoma and ependymoma, adjuvant radiotherapy after surgery helps improve local control,^52-54^ while for other histologies, such as pure germinoma, or nongerminomatous germ cell tumor, radiotherapy can be a definitive local therapy after induction chemotherapy^55-59^ with these patients achieving excellent long-term outcomes. However, many aggressive CNS histologies, such as high-grade gliomas or embryonal tumor with multilayered rosettes (ETMR), carry a poor prognosis even with aggressive treatment, including radiotherapy.^60-62^

As many pediatric patients are long-term survivors after radiotherapy for CNS malignancies, there can be significant long-term toxicities, including changes in memory or neurocognitive function, hearing loss, endocrinopathies, decreased growth, strokes, and secondary neoplasms.^63-67^ Therefore, delivering conformal radiotherapy while decreasing dose to normal organs is paramount to the long-term health for pediatric patients with brain tumors. Advances in external beam radiotherapy with the development of intensity-modulated radiotherapy, volumetric-modulated arc therapy, and proton radiotherapy have greatly improved normal tissue sparing while maintaining excellent local control rates.^68-71^ In particular, proton radiotherapy decreases the integral dose to normal brain due to the advantageous dose profile as compared to photons, and many studies have demonstrated improvement in various short-term and long-term toxicities when using proton radiotherapy.^72-76^ Despite these advances, side effects remain and there is significant interest in novel radiation techniques, such as FLASH-RT, and their potential to improve treatments for pediatric patients with CNS tumors.

## The potential of FLASH-RT for pediatrics

A summary of key preclinical FLASH studies relevant to pediatric tumors described in this review is provided in Table 1.

**Table 1:** Summary of key publications described in this review, with associated preclinical models, main findings, and relevance for pediatric applications

Study	Preclinical model/patient population	Radiation	Key findings	Relevance for pediatrics
Montay-Gruel et al., 2017,^8^ 2019^25^; Simmons et al., 2019^10^ ^a^	Adult C57BL/6J mice, whole brain	Single-dose electron, 10-30 Gy; FLASH (>100 Gy/s) vs. conventional (0.1 Gy/s)	FLASH-RT preserved cognitive function and reduced neurotoxicity	First demonstrations of neuroprotection by FLASH-RT in the brain; supports potential translation to pediatric brain sparing
Alaghband et al., 2020^9^	Juvenile (3 wk) C57BL/6J mice, whole brain	Single-dose electron, 8 Gy; FLASH (4 × 10^6^ Gy/s) vs. conventional (0.1 Gy/s)	FLASH reduced cognitive dysfunction	First demonstration of FLASH sparing in juvenile brain; relevant for medulloblastoma
Williams et al., 2022^13^	Neonatal (P11) Sprague Dawley rats, whole brain	Single-dose proton (plateau), 5-8 Gy; FLASH (100 Gy/s) vs. conventional (1 Gy/s)	FLASH reduced neurotoxicity; delayed/milder weight loss and distinct dopaminergic responses vs. adults (in previous study)	Shows FLASH neuroprotection in developing brain using protons; highlights age-dependent responses
Allen et al., 2023^81^	Juvenile (3 wk) C57BL/6J mice, whole brain	Hypofractionated electron, 2 × 10 Gy; FLASH (6 × 10^6^ Gy/s) vs. conventional (0.1 Gy/s)	FLASH preserved cognition and synaptic/vascular integrity with reduced neuroinflammation	First hypofractionated FLASH study in juvenile brain; relevant for pediatric medulloblastoma
Limoli et al., 2023^88^	Adult C57BL/6J mice, whole brain	Fractionated electron, 10 × 3 Gy, FLASH (2 × 10^6^ Gy/s) vs. conventional (0.1 Gy/s)	FLASH preserved cognition in fractionated regimen	Extends FLASH-RT to clinically relevant fractionation; potential applicability to pediatric protocols
Padilla et al., 2024^103^	Juvenile (6 wk) B6(Cg)-Tyrc-2J/J mice, DIPG orthotopic model, hindbrain	Single-dose electron, 15 Gy, FLASH (90 Gy/s) vs. conventional (0.03 Gy/s)	Equivalent tumor control for FLASH and conventional RT; immune response differences over time	Pediatric-relevant DIPG FLASH study; shows FLASH alters immune timing without affecting tumor control
Ni et al., 2025^92^	Juvenile (6-8 wk) C57BL/6J mice; genetically engineered MB or orthotopic glioma, hindbrain	Single-dose proton (plateau), 10 Gy, FLASH (80-135 Gy/s) vs. conventional (0.6-0.85 Gy/s); CAR-T	FLASH enhanced CAR-T efficacy, proinflammatory macrophage polarization, improved survival	Demonstrates immune modulation and therapy sensitization from FLASH-RT in pediatric brain tumors
Ruan et al., 2021^109^	Young adult (9-10 wk) vs. aged (30-31 wk) C3H mice, whole abdomen	Single-dose electron, 11-12.5 Gy, FLASH (2-6 × 10^6^) Gy/s vs. conventional (0.25 Gy/s)	Reduced microbiome disruption from FLASH; age- and pulse-dependent intestinal sparing	Shows age-dependence in FLASH sparing; potential importance for pediatric GI toxicity considerations
Iturri et al., 2023^27^	Young adult (7 wk) Fischer 344 rats, naive and RG2 glioma-bearing, unilateral brain	Single dose proton (plateau), 15 or 25 Gy, FLASH (260 Gy/s) vs. conventional 4 Gy/s	Supplemental O₂ in anesthesia abolished FLASH sparing; impacted anti-tumor immune response	Highlights anesthesia and oxygen effects; critical for pediatric RT protocols
Bourhis et al., 2019^46^	Single patient, 75-year-old with multiresistant CD30+ T-cell cutaneous lymphoma	Electron, 15 Gy single fraction, FLASH (90 ms delivery)	First human FLASH-RT treatment; complete tumor response with minimal skin toxicity (no direct conventional comparison)	Demonstrated feasibility and safety of electron FLASH-RT in humans; proof-of-concept for clinical translation in superficial targets
Daugherty et al., 2022 (FAST-01 Trial)^47^	10 Adult patients (27-81 years) with painful extremity bone metastases	Proton, 8 Gy single fraction, FLASH (≥40 Gy/s)	First in-human trial; evaluated the feasibility of a clinical workflow for proton FLASH-RT	Established feasibility of clinical proton FLASH workflow; supports expansion to other indications, including pediatric bone or sarcoma RT

a This table highlights selected examples of FLASH studies. A more comprehensive summary of FLASH data can be found in various reviews,^4^^,^^6^ or full experimental parameters can be found in the FLASH study database,^131^ available at: https://exuberant-beak-513.notion.site/354aa5e33bad4771b65168bb613c5768? v=92b484f62472447aaaf00a4c1c31797e.

### Preclinical basis for FLASH-RT in pediatric CNS tumors

One of the most studied preclinical models in FLASH research has been the rodent brain, with and without the presence of tumors.^77^^, ^^78^ The long-term cognitive performance of mice after whole-brain irradiation has been found in multiple studies to be spared by FLASH compared to conventional irradiation.^8^^,^^10^^,^^25^ While most studies to date have used high-energy electron beams, comparable effects have been demonstrated using other beam types including synchrotron X-rays^79^ and proton beams.^80^ Besides behavioral endpoints, mitigation of multiple other parameters of brain injury known to be induced by irradiation have been demonstrated with FLASH, including multiple markers of neuroinflammation, neurogenesis, neuronal ultrastructure, and electrophysiology.^8^^,^^10-12^^,^^25^^,^^79^^,^^81^ The robustness of these findings has been confirmed through multi-institutional studies cross-validating first the dosimetry and then the biological reproducibility of FLASH brain irradiation.^82-84^

Concurrent with sparing normal brain toxicity, FLASH-RT has been shown in several orthotopic and subcutaneous rodent brain tumor models to have similar anti-tumor efficacy as conventional irradiation in both single and multi-fraction regimens.^80^^,^^85-87^ One study observed brain sparing from FLASH-RT in fractionated irradiation regimens, including conventional fractionation (30 Gy in 10 fractions of 3 Gy),^88^ which would be highly relevant for clinical translation; however, many studies suggest that larger hypofractionated doses (∼10 Gy) are necessary to achieve FLASH sparing.^81^^,^^85^^,^^89^ The observed dose threshold could be due to a lack of induced toxicities when smaller doses are delivered. Recent studies investigating split doses, delivering a single, larger dose with short time gaps between 2 or more dose pulses, showed varied results, with some reporting a loss of the FLASH effect, while others maintained tissue sparing albeit at a reduced level.^90^^,^^91^ For clinical translation, we need to clearly identify any potential dose thresholds. Importantly, the developing brain is more sensitive to the adverse effects of irradiation, particularly in terms of late effects. Thus, it is important to evaluate whether the FLASH effect is maintained in models of pediatric brain irradiation. To date, only 3 studies have shown FLASH sparing after whole brain irradiation in juvenile or neonatal mice and rats.^9^^,^^13^^,^^81^

In summary, while many important questions remain, there are ample preclinical data so far to suggest the promise of FLASH-RT for improving the therapeutic index of radiotherapy for pediatric CNS tumors, an indication for which radiotherapy is both crucial and in crucial need of improvement.

### FLASH-RT is a new frontier for brain tumor treatment

Brain tumors including high-grade glioma and medulloblastoma are the most common solid tumors affecting children and adolescents, posing significant treatment challenges. Even with intensive multimodal therapies—including surgery, chemotherapy, and radiation—patients with high-risk glioma and medulloblastoma often face poor prognoses. Moreover, survivors frequently endure long-term cognitive deficits due to the neurotoxic effects of traditional treatments such as craniospinal radiotherapy. FLASH-RT minimizes neurocognitive side effects in preclinical models,^25^ providing durable protection against cognitive decline while retaining efficacy against aggressive brain tumors. These collective efforts underscore FLASH-RT’s potential for brain tumor therapy, particularly where preserving cognitive function is paramount.

### FLASH-RT acts as an immune modulator in brain tumor

While immunotherapies like chimeric antigen receptor (CAR)-T cells have revolutionized blood cancer treatment, their effectiveness in solid tumors including brain tumor has been hindered by the suppressive nature of the tumor microenvironment (TME). Tumor macrophages are often the major source of immune suppression in brain tumor, representing a critical target for overcoming immunotherapy resistance. A recent study shows that FLASH-RT may address both the neurotoxicity and immune-resistance issues, presenting it as a novel adjunct to CAR-T cell therapy in medulloblastoma.^92^

RT could be considered as an “in situ vaccination” treatment to stimulate anti-tumor immunity, as it causes tumor cell lysis to release tumor antigens that are recognized by immune cells.^93^ However, the impact of standard radiotherapy on macrophage phenotypes seems to be conflicting, which depends on dose, fractionation, location, and cancer types,^94-100^ posing challenges for combining immunotherapy with radiotherapy. In addition to its tissue-sparing capabilities, FLASH-RT also appears to modulate the immune response in ways distinct from conventional radiation. FLASH-RT preserves peripheral immune cell populations, potentially contributing to its reduced toxicity profile.^101^ FLASH-RT also suppresses immune checkpoint protein expression on tumor cells, thereby enhancing tumor immunogenicity and responsiveness to immune therapies.^102^ Interestingly, FLASH and standard radiotherapy stimulate type I interferon responses with different kinetics and in distinct immune cell populations in diffuse midline glioma,^103^ collectively suggesting that FLASH-RT induces a unique immunological “rhythm” compared to standard radiotherapy, potentially creating new opportunities for optimizing immunotherapy timing and efficacy. Together, these insights position FLASH-RT as a dual-function modality—both protecting normal brain tissue and modulating immune responses to enhance therapeutic impact.

Another reported effect is that FLASH-RT may reprogram lipid metabolism to modulate macrophage activity via suppressing oxidation of lipoprotein and activation of PPAR-γ,^92^ a driver of immunosuppressive macrophage polarization. Consistent with these findings, FLASH-RT induces reduced oxylipin formation compared to standard radiation.^104^ The metabolic shift is linked to improved GD2 CAR-T cell infiltration and functionality,^92^ suggesting that FLASH-RT may reprogram the TME in ways that favor immune engagement. Likewise, FLASH-RT sensitizes tumors to GD2 CAR-T cell immunotherapy in preclinical models of medulloblastoma and glioma.^92^ Considering the safety and promising efficacy of GD2 and B7-H3 CAR-T cells, recently explored by intravenous and intracranial injection in patients with diffuse midline glioma,^105-107^ FLASH-CAR radioimmunotherapy is a promising strategy in pediatric brain tumors.

### FLASH radiotherapy in developing embryos and fetuses

Radiation exposure during embryonic and fetal development is known to be detrimental, often fatal, or associated with long-term developmental abnormalities. Survivors of in utero radiation are also at higher risk of leukemia and other cancers.^108^ Therefore, it is critical to investigate the effects of FLASH-RT on embryos and fetuses, particularly in immunocompetent rodent models. These studies could yield valuable insights into the differential impact of FLASH versus conventional radiation on various stages of development and the stem cell populations essential for growth and tissue regeneration. Clinically, however, doses to the uterus during radiation therapy are generally kept well below 1 Gy, potentially limiting the achievable FLASH effect in utero.

### A new path ahead

By curbing the neurotoxic side effects traditionally associated with pediatric cranial radiotherapy while simultaneously enhancing immune responsiveness, FLASH-RT opens a new avenue in pediatric neuro-oncology. However, many questions remain. The precise biological pathways that distinguish FLASH from conventional radiotherapy—especially those involving lipid regulation—warrant further investigation. The effects of FLASH-RT on other immunosuppressive cell types, such as regulatory T cells and tumor-associated fibroblasts, remain unclear. Given the brain’s high lipid content (constituting approximately 50% of its dry weight) and the abundance of lipid-laden macrophages in the brain tumor, FLASH-RT may prove particularly well-suited to CNS malignancies. Future work must also focus on refining beam parameters, timing, and dosage to fully exploit the synergy between FLASH-RT and CAR-T cell therapy as well as other immunotherapeutic approaches such as vaccines, immune checkpoint inhibitors, and oncolytic viruses. Clinical trials are essential to confirm these findings in patients and to determine the long-term safety profile of this promising therapeutic strategy. The convergence of high-precision radiation and cutting-edge immunotherapy could be the start of a transformative era in the treatment of pediatric brain tumors.

## Challenges and risks for translation

Despite the potential of FLASH-RT, particularly for pediatric neuro-oncology, significant challenges remain in translating preclinical promise into clinical practice. While early-phase trials have demonstrated the feasibility of delivering ultra-high dose rates, no clinical study has yet reported direct comparisons between FLASH and conventional radiotherapy in humans, and no study to date has included pediatric patients. As we move toward clinical adoption, key questions remain for pediatrics.

### Age-dependent biological responses

One important consideration is how age-related biological differences might influence treatment response. Few studies have directly compared the FLASH effect across different ages in animal models. Ruan et al.^109^ observed differences in relative crypt survival between FLASH and conventional radiotherapy in mice aged either 9-10 weeks or 30-31 weeks, particularly in how the FLASH effect depends on dose rate and pulse structure for each age-group. However, more studies are needed to clarify any age-dependence of FLASH-RT. Notably, the protective effects of FLASH-RT have been shown to depend on factors that may vary with age, such as tissue oxygenation.^25-27^^, ^^29^ Physiological differences between children and adults—such as higher metabolic rates, increased respiratory rates, and greater cardiac output per kilogram—may alter tissue oxygen dynamics, potentially impacting the biological effects of FLASH. Since the mechanism underlying FLASH sparing remains unknown, it is difficult to predict whether the relevant biological processes might behave differently in pediatric patients.

A leading hypothesis for the FLASH sparing effect is that FLASH-RT rapidly depletes oxygen, outpacing resupply from the blood and inducing a transient hypoxic, radioresistant state in normal tissue. While it remains unclear whether this can fully account for the FLASH effect, preclinical studies have shown that FLASH-RT does cause oxygen depletion in tissue.^31^ It is possible that, in pediatric patients, faster circulatory and respiratory dynamics may accelerate reoxygenation,^110^^,^^111^ potentially resulting in reduced oxygen depletion, or higher dose rates required to achieve the same effect. This theory also assumes that tumors, often already hypoxic, experience less of a change in radiosensitivity than normal tissue, allowing for selective sparing of healthy tissue. However, many pediatric tumors are thought to be less hypoxic than adult tumors,^112^ which could undermine this differential effect or even preferentially spare the tumor. More broadly, age-related differences in the TME may influence radiochemical processes involved in the response to FLASH-RT.^113^ While this remains speculative, it could have important implications on clinical implementation of FLASH-RT for pediatric patients.

In addition to oxygen dynamics, other biological factors may influence how children respond to FLASH-RT. For example, FLASH-RT alters the temporal dynamics of dose delivery compared to conventional radiotherapy, reducing the volume of blood and circulating immune cells irradiated during treatment.^101^ These effects are likely to vary between children and adults due to differences in vascular architecture, blood flow patterns, and relative size between the irradiation field and body size. Moreover, several studies suggest that the immune system plays a critical role in mediating FLASH sparing effects.^114^ Importantly, pediatric response may differ from that of adults. Children are born with immature, innate and adaptive immune systems, with a predominance of naive T and B cells, less immunological memory, and developing innate immune responses (such as functionally immature macrophages). Their immune system matures with age and at old age, the immune system declines resulting in dysregulated adaptive immunity.^115^ These differences could influence the immunomodulatory effects of FLASH-RT, including macrophage reprogramming and T-cell infiltration, potentially altering both normal tissue sparing and anti-tumor immune responses compared to adults. Ultimately, to predict whether the same protective benefits will be preserved in pediatric patients, we need to understand the underlying mechanism of FLASH sparing. Careful investigation is needed before widespread clinical translation in this population.

The clinical use of FLASH-RT in pediatric patients will be the ultimate test of its safety and efficacy. However, as discussed, numerous unknowns remain regarding its effects on immature tissues. These must be addressed in preclinical models before initiating FLASH-RT treatments in children. While some evidence suggests neuroprotective effects in pediatric rodent models,^9^^,^^13^^,^^81^ the behavioral assays used in mice are crude and do not capture the full spectrum of neurocognitive function. In contrast, human neurocognitive assessments are much more sophisticated and capable of evaluating a wide range of psychological and cognitive domains.

Some studies have also explored effects on gastrointestinal crypt cells,^109^ but key gaps remain—particularly regarding growth in bone, soft tissue, and organs such as the heart, lungs, liver, and kidneys. In children, these organs must not only function adequately but also grow proportionally with the patient. Therefore, both functional and developmental outcomes must be evaluated before any clinical translation for pediatric patients.

Beyond inherent physiological differences, clinical factors unique to pediatric radiotherapy must also be considered. For example, anesthesia is often required for very young children undergoing treatment. A variety of anesthetic agents—including propofol, dexmedetomidine, midazolam, and ketamine—are used in clinical practice, without a standardized consensus on choice or protocol.^116^ These agents can influence heart rate and respiratory function,^116^^,^^117^ which in turn may impact tissue oxygenation levels. Preclinical studies have demonstrated that different types of anesthesia can significantly alter oxygen availability in tissues,^118^ and that supplemental oxygen during anesthesia may even eliminate the FLASH effect.^27^ This issue is particularly relevant in pediatrics, where approximately 15% of patients require full or partial anesthesia during radiotherapy, rising to nearly 100% in children under 3 years old.^117^ Although the fast delivery of FLASH-RT could reduce treatment times, potentially helping to mitigate the need for anesthesia in some pediatric patients, its overall impact may be limited, since beam-on time only constitutes a small portion of the total setup and treatment duration. Given these complexities, understanding the interplay between anesthesia and FLASH-RT is critical before applying this treatment modality in pediatric populations.

### Delivery techniques and constraints

Translating any novel therapy or technology is not without challenges or risks. In the field of FLASH-RT, with the first in-human clinical trials already completed in the adult setting, many areas of risk have been identified. There are many considerations that are generally true and not unique to pediatric patients. For example, the technological evolution of FLASH-RT machines creates technological gaps therefore necessitating the development of novel and fast quality assurance systems and beam monitors.^119^ These gaps also, in the near term, hinder inter-institutional FLASH-RT clinical trial design or remote clinical trial credentialing and auditing. This, and many more areas, are identified as challenges and risks for FLASH-RT clinical translation that impact both adult and pediatric populations.^119^ However, in the pediatric population, certain considerations may have special importance and impact.

Most pre-clinical studies demonstrate a FLASH-induced normal tissue sparing effect for larger fraction sizes, indicating a possible preference for hypofractionated dose schemas.^81^^,^^85^^,^^89^ However, in a pediatric population, for curative intent at standard dose rates, hypofractionation is rarely used due to the reduced normal tissue sparing with increasing fraction size. Balancing the normal tissue sparing effects of both FLASH-RT and fractionation is necessary for optimal translation into pediatric disease. It is important to note that some data show a preservation of the FLASH effect for conventional fraction sizes,^88^ while others show a reduction or loss of the FLASH effect if the doses are split or the time between doses exceeds a few seconds. If fractionation can be maintained, this synergy between FLASH-RT and fractionation would be ideal.

Both conventional dose rates and FLASH dose rates have associated toxicity, albeit reduced for FLASH, depending on the amount of radiation dose. Furthermore, it is established that adult normal tissue tolerances are not always aligned with pediatric normal tissue tolerances.^120^ Most pre-clinical data of the FLASH effect are gathered in adult animal models, highlighting a further need for studies in juvenile animals. Several groups reported neurocognitive sparing, of particular importance in a growing and developing brain, in juvenile pre-clinical models. Allen et al.^81^ show a neurocognitive sparing effect for juvenile mice with hypofractionation and FLASH irradiation. Alaghband et al.^9^ show neuroprotective effect from FLASH-RT in juvenile mice as a function of time post irradiation, demonstrating a clearer effect at 4-months than at 2-months. Williams et al. show neurocognitive sparring in juvenile rats as a function of dose and dose rate.^13^ However, evidence of sparing outside of the brain in juvenile models is lacking. Given adult and pediatric normal tissues have different tolerance, their potential sparing effects from FLASH-RT may also be different.

Another normal tissue toxicity of particular importance for pediatric patients is the risk of secondary radiation-induced malignancies.^121^^,^^122^ Because the FLASH effect de-emphasizes the importance of only physical dose conformality as the primary way of sparing healthy tissues, special care must be taken not to significantly increase the integral dose, particularly in pediatric populations. There are limited models studying the induction of secondary malignancy for radiation, and, at the time of writing of this article, no publication compares radiation-induced secondary malignancies for FLASH and conventional dose rates. Given the lack of confirmatory data demonstrating the secondary malignancy risks of conventional vs. FLASH radiation in animal or analytical models, the impact is mostly unknown. While few pre-clinical studies investigated longer term effects (typically in the order of a year),^2^^,^^25^^,^^44^ we lack long-term follow-up data (both preclinical and clinical) as FLASH-RT is a relatively new modality, making it difficult to assess the risk of secondary malignancies or other late effects. Understanding long-term effects is an unknown but important consideration, uniquely critical in the pediatric setting.

## Principles of clinical trial design for pediatrics and FLASH

As discussed above, many challenges remain. For clinical translation in pediatric patients, potential differences in the FLASH effect between adult and pediatric populations have to be evaluated carefully. In particular, the FLASH effect must be robustly demonstrated without causing long term complications. Moving forward, comprehensive preclinical studies across the pediatric age spectrum (eg, juvenile models) are essential to confirm that FLASH-RT’s normal tissue sparing holds true for developing organs and that no unique adverse effects emerge in immature systems. Such evidence will be key to satisfying ethical and regulatory bodies (Institutional Review Boards [IRBs], the FDA, etc.) that FLASH-RT can be attempted in children with appropriate safeguards. In short, a strong foundation of both adult clinical data and pediatric-focused preclinical data is a prerequisite for pediatric FLASH trials.

### Establishing safety before clinical use in children

Before considering the use of FLASH-RT in pediatric patients, we must rigorously evaluate its effects on developing tissues across all stages of growth to ensure that no catastrophic or unforeseen adverse events occur. As with any novel therapy, FLASH-RT will first need to be tested in adults. The first-in-human trial of proton FLASH, FAST-01 (FeAsibility Study of FLASH Therapy for the Treatment of Symptomatic Bone Metastases), was conducted under an FDA Investigational Device Exemption (IDE) in adults to establish baseline safety, workflow feasibility, and efficacy.^48^ The FAST-01 pilot clinical trial tested the effects of FLASH-RT on a variety of normal tissues in the limb while avoiding critical organs. The results demonstrated clinical feasibility, efficacy, and supported the hypothesized safety: no unexpected acute toxicity was observed. All adverse events were mild and consistent with those expected from conventional radiation. However, the trial did not include a comparative conventional-dose-rate treatment arm. The positive outcomes, coupled with an absence of significant normal tissue injury, informed the next steps in clinical development.

A second adult trial, Flash for Thoracic Bone Metastases (FAST-02), also under an FDA IDE, recently completed enrollment.^49^ FAST-02 expanded the application of proton FLASH to deep-seated tumors by treating 10 patients with painful bone metastases in the thorax (eg, ribs or chest wall) at 8 Gy × 1 delivered at >40 Gy/s, also exposing thoracic organs to FLASH doses. The 2 FAST trials illustrate the stepwise approach: first confirm safety in adult extremities, then extend to adults with tumors near vital organs. This de-risking in adults is an ethical imperative before pediatric use. All findings from these adult studies must be carefully analyzed and shared to inform pediatric trial design. Importantly, when moving into pediatric clinical trials, the standard principles of trial design must apply. A Phase I (safety and dose-finding) trial is essential. However, enrolling children in Phase I trials demands a prospect of therapeutic benefit. As such, initial trials will likely focus on children with recurrent or incurable disease, where potential benefit outweighs risk and with palliative goals, or patients where current standard of care treatments can be delivered at FLASH dose rates.^78^

### Ideal first pediatric trials and indications

Early pediatric applications of FLASH-RT will likely focus on patients with recurrent or incurable cancers for whom conventional therapies offer poor outcomes. There is strong precedent for offering experimental treatments (under FDA investigational protocols) to children with bleak prognoses and no effective alternatives, eg, novel chemotherapies under investigational new drug (IND) trials for relapsed leukemias or diffuse midline gliomas (DMG). A parallel logic can be applied to FLASH-RT. The most immediately compelling pediatric indications are those that *either* leverage the adult FLASH experience in analogous disease sites *or* are pediatric-specific diseases with extraordinary preclinical support.

One promising avenue is refractory or metastatic sarcomas in the extremities or thorax—scenarios that mirror the FAST-01 and FAST-02 adult trials. For instance, recurrent osteosarcoma or Ewing sarcoma lung metastases in adolescents could be candidates. These tumors are often resistant to conventional palliative radiation doses but show better outcomes with stereotactic radiosurgery or high-dose fractions.^123^ Using FLASH-RT to deliver an ablative dose to a metastatic lesion could improve symptomatic relief or local control, while minimizing collateral damage to normal tissue. Critically, treating lesions in the lung or extremities in a child would build directly on adult safety data for those anatomic sites. Highly conformal techniques targeting only gross tumor volumes are preferable in pediatric patients to minimize the risk of unanticipated normal tissue toxicity. In this setting, FLASH proton radiotherapy may be the optimal modality.

While the greatest long-term clinical benefit of FLASH might eventually be in protecting sensitive organs like the brain, the initial pediatric CNS application will be cautious and likely in the palliative setting. One compelling example is recurrent DMG—a fatal brainstem tumor unique to children. These tumors have a dismal prognosis (median survival ∼9-12 months, with <1% 5-year survival^124^) and treatment options are limited.^125^ Standard radiotherapy (54 Gy in 30 fractions) is the only effective treatment to transiently improve symptoms, but nearly all patients progress within months of completing it. At relapse, some children receive re-irradiation (e.g. ∼20-30 Gy in 10-14 fractions) which can provide a few additional months of symptom relief and survival. However, cumulative toxicity to brain tissue is a serious concern.^126^ In this dire context, FLASH-RT could be a game-changer and a Phase I FLASH trial for children with recurrent DMG under an FDA IDE could potentially be justified with the rationale to attempt a higher radiation dose or second course of radiation to the tumor with reduced risk of brainstem injury. Such a trial would enroll only children who have exhausted standard therapies. An early signal of benefit—tumor control or neurological improvement—would be immensely valuable given the otherwise grave outcomes. The extraordinary need in DMG and robust multi-species preclinical data demonstrating neurocognitive preservation and maintained tumor control could potentially provide ethical and regulatory justification.

Additional compelling initial indications for FLASH may also include curative pediatric brain tumors that require modest-to-high doses of radiation and occur near critical dose-limiting normal structures such as brainstem or spinal cord. Such histologies include ependymomas, high-grade gliomas of the spine, and optic pathway gliomas. Lastly, a significant number of pediatric brain tumor histologies that are curable at initial diagnosis carry extremely poor prognoses at recurrence, for example, medulloblastoma and ependymoma. FLASH offers exciting translational opportunities in these challenging settings.

In summary, ideal first pediatric FLASH trials would fall into 2 categories: (1) those extrapolating from adult trials, where FLASH might palliate disease with less toxicity; and (2) those targeting pediatric-specific lethal diseases with strong preclinical backing. In both scenarios, initial use would be palliative or salvage in nature, involving patients with limited life expectancy and no curative options. This aligns with standard pediatric trial ethics: higher-risk experimental therapies are first offered to patients who are incurable with existing treatments, in hopes of palliation or extension of life. Early pediatric FLASH trials will likely use single-fraction or very hypofractionated regimens targeting only gross tumors, with meticulous normal tissue dose constraints, to minimize any unforeseen toxicity. These early trials must also include outcome metrics for Quality of Life, pain, and other metrics for symptom response to know if FLASH is indeed better than standard palliative radiotherapy. Only after safety is confirmed in these limited contexts would expansion to broader or curative pediatric indications be entertained.

### Toward curative and broader use in pediatrics

As experience grows and evidence accumulates, FLASH-RT could be introduced into curative settings. As with proton therapy, the transition from palliative to curative use must be accompanied by meticulous documentation of both acute and late toxicities. Disease-specific outcomes and morbidity endpoints will both be necessary. For FLASH-RT to be adopted as part of the pediatric oncology treatment arsenal, it must either reduce treatment-related morbidity or improve disease control, or ideally, both.

Another potential benefit for Flash RT is the great potential for hypofractionation. Quick treatments with low morbidity result in as less time receiving treatment and more time with family and friends which is a major objective in palliative care for children improving their quality of life. In addition, given goals of the treatment and late effects may be less of an issue, and treatment planning may be sped up with wider fields and less need for anesthesia and the nutritional challenges daily anesthesia can pose in a child.

The acceptance of proton therapy provides a useful parallel. Its adoption was based on evidence showing equivalent disease control and reduced morbidity compared to conventional radiotherapy.^127^ Today, proton therapy is the preferred radiation modality by most clinicians for treating certain curable pediatric solid tumors in the United States and many other countries with the necessary infrastructure.^128^^,^^129^

## Summary and outlook

The future of FLASH-RT in pediatric oncology is full of promise, but significant preclinical and adult clinical groundwork remains. Detailed studies are required to determine appropriate dosing and fractionation schedules, assess effects on tissue growth and function, and identify potential toxicities.

Initial pediatric clinical trials should be Phase I studies either in scenarios where current standard of care can be directly replaced 1:1 with FLASH-RT, or in children with recurrent or otherwise untreatable disease, focusing on palliative outcomes. The FAST-01 and FAST-02 trials offer a blueprint for technology implementation and regulatory oversight. If such studies demonstrate safety and benefit, Phase II trials will follow. In the event of compelling Phase II results—particularly if FLASH-RT shows clear reductions in toxicity while maintaining disease control—Phase III randomized trials may be deemed unethical due to a lack of equipoise, similar to what occurred with proton therapy in pediatrics.^130^

FLASH-RT is especially compelling for pediatric applications, given its potential to spare developing tissues. Current and future adult clinical trials will be essential to extrapolate potential benefits for pediatric patients. Careful demonstration of maintaining the FLASH effect across ages in animal models, with special considerations for the differences between adult and pediatric metabolism, tissue growth, immune system and tumor properties, should be conducted to provide confidence before widespread clinical use in pediatric patients. Simultaneously, FLASH-RT must show at least equivalent tumor control to existing standards, such as proton therapy. We also need better understanding of tumor- and tissue-specific dose-response relationships in the FLASH-RT setting.

While preliminary clinical results in adults have been reassuring in limited scenarios, the depth and breadth of clinical experience with FLASH-RT remain limited. Future pediatric trials will require rigorous collection of morbidity and efficacy data, as well as biologic correlates to elucidate underlying mechanisms. Significant investment in infrastructure, resources, and technology will be needed to incorporate FLASH-RT into routine clinical workflows.

## Lay summary

Pediatric patients present unique challenges in radiotherapy due to their generally favorable clinical prognosis and long life expectancies. Advances in treatment techniques have improved outcomes, however, the risk of late effects, including neurocognitive decline remains a major clinical concern in pediatric oncology. FLASH radiotherapy (FLASH-RT), a technique delivering radiation in a few milliseconds, has emerged over the past decade as a potential game-changer, promising to spare normal tissue while preserving tumor control. We examine the potential and risks of FLASH-RT for pediatric cancers, carefully assessing the benefits and risks to guide safe integration of FLASH-RT into routine pediatric cancer care.
